# Crystal structure of *N*-(2-amino-5-cyano-4-methyl­sulfanyl-6-oxo-1,6-dihydropyrimidin-1-yl)-4-bromo­benzene­sulfonamide di­methyl­formamide monosolvate

**DOI:** 10.1107/S2056989015018903

**Published:** 2015-10-14

**Authors:** Galal H. Elgemeie, Reham A. Mohamed, Hoda A. Hussein, Peter G. Jones

**Affiliations:** a Chemistry Department, Faculty of Science, Helwan University, Cairo, Egypt; b Photochemistry Department, National Research Center, Dokki, Cairo, Egypt; c Institut für Anorganische und Analytische Chemie, Technische Universität Braunschweig, Postfach 3329, D-38023 Braunschweig, Germany

**Keywords:** crystal structure, pyrimidine, bromo­benzene­sulfonamide, hydrogen bonding

## Abstract

The title compound, C_12_H_10_BrN_5_O_3_S_2_·C_3_H_7_NO, displays an almost planar amine group. The inter­planar angle between the rings is 31.72 (6)°. The residues are associated into ribbons parallel to [110] by three classical hydrogen bonds; one from each amine H_amine_ to O_DMF_ and one from NH_amide_ to O_oxo_. Adjacent ribbons are connected by translation parallel to the *c* axis by a ‘weak’ hydrogen bond H_meth­yl_⋯O_sulfon­yl_ to form a layer structure parallel to (1-10), while a further contact H_bromo­phen­yl_⋯O_sulfon­yl_ connects the residues in the third dimension.

## Abstract 2

In the title compound compound, the phenyl and pyrimidine rings are inclined to one another at 31.72 (6)°. The residues are associated into ribbons parallel to [110] by three classical hydrogen bonds. Adjacent ribbons are connected by translation parallel to the *c* axis by a ‘weak’ hydrogen bond to form a layer structure parallel to (1-10), while a further contact connects the residues in the third dimension.

## Chemical context   

We are conducting studies directed towards exploring the synthetic potential of dimethyl *N*-cyano­imido-*S*,*S*-dimethyl-di­thio­carbonate and other ketene di­thio­acetals for synthesizing new classes of anti­metabolites (Elgemeie & Mohamed, 2014 ▸; Elgemeie *et al.*, 2007 ▸, 2009 ▸). We have recently reported various successful approaches to the synthesis of mercapto­pyrimidines by the reaction of this compound with active methyl­ene functions (Elgemeie & Sood, 2001 ▸; Elgemeie *et al.*, 2003 ▸). In an extension of this work, we describe a one-pot synthesis of *N*-(2-amino-5-cyano-4-(methyl­thio)-6-oxopyrimidin-1(6*H*)-yl)-4-bromo­benzene­sulfonamide (I) by the reaction of dimethyl *N*-cyano­dithio­imino­carbonate with *N*′-(4-bromo­phen­yl)sulfonyl-2-cyano­ethane­hydrazide. The chemical nature was proposed on the basis of elemental analysis and spectroscopic data and its X-ray structure determination was undertaken to confirm the nature of the product. We have recently presented the structure of a related pyrimidine (Elgemeie *et al.*, 2015 ▸).
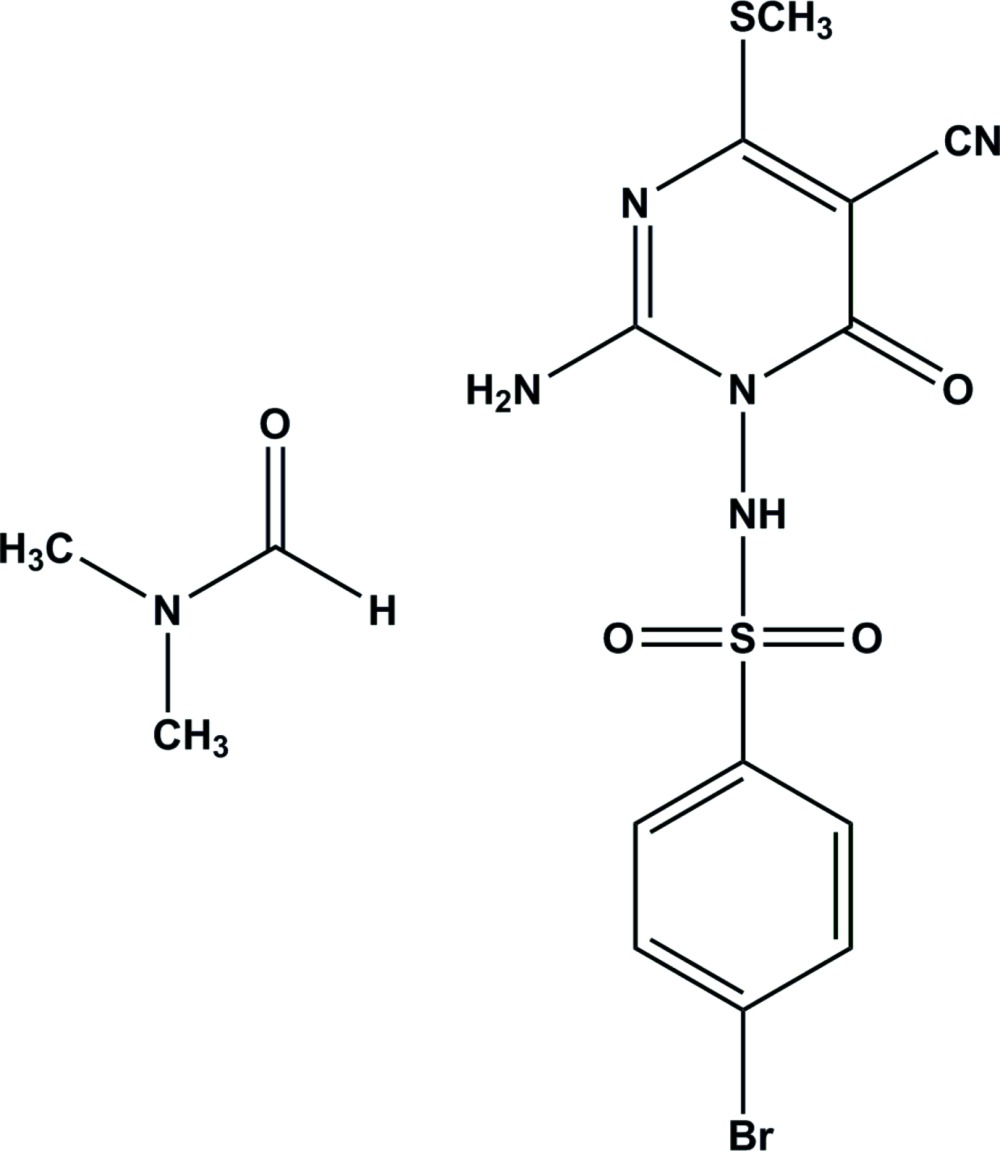


## Structural commentary   

**Figure 1:**
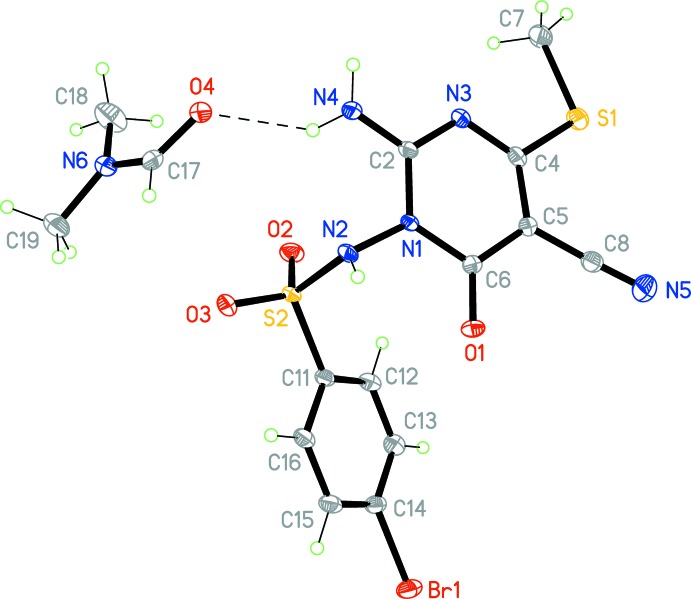
The formula unit of compound (I)·DMF in the crystal. Displacement ellipsoids correspond to 50% probability levels. The hydrogen bond H03⋯O4 is drawn as a thin dashed line.

The structure of the title compound, which proved to be the di­methyl­formamide solvate (I)·DMF, is shown in Fig. 1 ▸. The ring systems are as expected almost planar, with r.m.s. deviations of 0.002 Å for the phenyl and 0.04 Å for the pyrimidine ring. The substituent atoms N4 and S1 deviate significantly from the pyrimidine plane [by 0.199 (2) and 0.257 (2) Å respectively, to opposite sides of the plane]. The inter­planar angle is 31.72 (6)°, and is also associated with the torsion angles C12—C11—S2—N2 88.10 (12), C11—S2—N2—N1 78.98 (11) and S2—N2—N1—C2 100.31 (12)°. The amino group at N4 is almost planar, with the nitro­gen atom lying just 0.035 (11) Å out of the plane of its substituents.

## Supra­molecular features   

**Figure 2:**
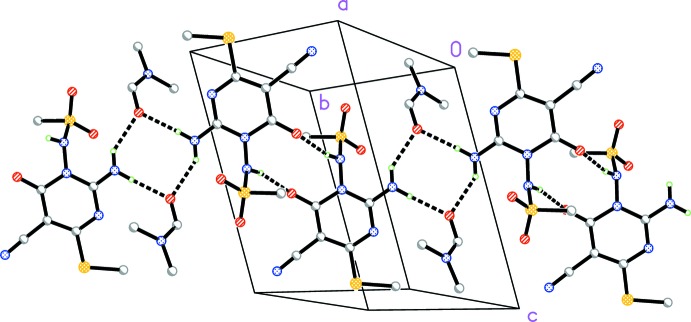
Packing diagram of compound (I)· DMF viewed perpendicular to (1
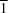
0). For clarity, only the *ipso* carbons of the bromo­phenyl groups are shown. Classical hydrogen bonds are indicated by dashed lines.

The components are associated into ribbons parallel to [110] (Fig. 2 ▸) by three classical hydrogen bonds (Table 1 ▸). Two of these, H02⋯O4(1 − *x*, −*y*, 1 − *z*) and H03⋯O4, involve the di­methyl­formamide oxygen atom and lead to the formation of inversion-symmetric rings of graph set 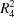
(8). The third hydrogen bond, H01⋯O1(2 − *x*, 1 − *y*, 1 − *z*), also forms inversion-symmetric rings, but of graph set 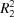
(10).

There are two short and acceptably linear C—H⋯O contacts that may be assumed to represent ‘weak’ hydrogen bonds; H7*B*⋯O3 connects neighbouring ribbons by translation parallel to the *c* axis, thus completing a layer structure parallel to (1
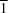
0), while H12⋯O2 connects the residues in the third dimension via the inversion operator (1 − *x*, 1 − *y*, 1 − *z*).

The bromine atom is involved in two secondary contacts: a halogen bond of 3.4582 (10) Å with O1(2 − *x*, 2 − *y*,1 − *z*) and a weak hydrogen bond of 3.05 Å from H17(*x*, 1 + *y*, *z*), with an angle of 124° at hydrogen. These inter­actions also connect the residues in the third dimension.

## Synthesis and crystallization   

Dimethyl *N*-cyano­imido-*S*,*S*-dimethyl-di­thio­carbonate (0.01 mol) was added to a stirred solution of *N*′-(4-bromo­phen­yl)sulfonyl-2-cyano­ethane­hydrazide (0.01 mol) in dry dioxane (50 mL) containing potassium hydroxide (0.01 mol) at room temperature. The reaction mixture was stirred for 30 min at room temperature; the precipitated solid was collected by filtration and crystallized from dimethyl formamide to give pale yellow crystals, m.p. 483–485 K, yield 85%.

## Refinement   

Crystal data, data collection and structure refinement details are summarized in Table 2 ▸. The NH hydrogens were refined freely. The methyl groups were refined as idealized rigid groups allowed to rotate but not tip. Other H were included using a riding model starting from calculated positions [C—H = 0.95–0.98 Å with *U*_iso_(H) = 1.5*U*_eq_(C) for methyl H atoms and 1.2*U*_eq_(C) for other H atoms].

## Supplementary Material

Crystal structure: contains datablock(s) I, global. DOI: 10.1107/S2056989015018903/lh5790sup1.cif

Structure factors: contains datablock(s) I. DOI: 10.1107/S2056989015018903/lh5790Isup2.hkl

Supporting information file. DOI: 10.1107/S2056989015018903/lh5790Isup3.cml

CCDC reference: 1430044

Additional supporting information:  crystallographic information; 3D view; checkCIF report

## Figures and Tables

**Table 1:** Hydrogen-bond geometry (, )

*D*H*A*	*D*H	H*A*	*D* *A*	*D*H*A*
N2H01O1^i^	0.80(2)	2.00(2)	2.7784(15)	165.5(19)
N4H02O4^ii^	0.86(2)	1.98(2)	2.8382(16)	177.9(19)
N4H03O4	0.85(2)	2.07(2)	2.8005(16)	143.1(18)
C12H12O2^iii^	0.95	2.46	3.4096(18)	173
C7H7*B*O3^iv^	0.98	2.45	3.2848(19)	143
C17H17Br1^v^	0.95	3.05	3.6686(15)	124

Symmetry codes: (i) 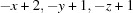; (ii) 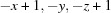; (iii) 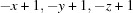; (iv) 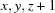; (v) 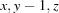.

**Table 2:** Experimental details

Crystal data	
Chemical formula	C_12_H_10_BrN_5_O_3_S_2_C_3_H_7_NO
*M*_r_	489.38
Crystal system, space group	Triclinic, *P* 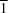
Temperature (K)	100
*a*, *b*, *c* ()	9.1107(4), 9.9911(4), 11.6498(6)
, , ()	96.482(4), 107.802(4), 99.322(4)
*V* (^3^)	981.33(8)
*Z*	2
Radiation type	Mo *K*
(mm^1^)	2.34
Crystal size (mm)	0.45 0.40 0.40

Data collection	
Diffractometer	Oxford Diffraction Xcalibur, Eos
Absorption correction	Multi-scan (*CrysAlis PRO*; Agilent, 2013 ▸)
*T*_min_, *T*_max_	0.824, 1.000
No. of measured, independent and observed [*I* > 2(*I*)] reflections	52130, 5844, 5306
*R*_int_	0.031
(sin /)_max_ (^1^)	0.722

Refinement	
*R*[*F* ^2^ > 2(*F* ^2^)], *wR*(*F* ^2^), *S*	0.027, 0.061, 1.06
No. of reflections	5844
No. of parameters	268
H-atom treatment	H atoms treated by a mixture of independent and constrained refinement
_max_, _min_ (e ^3^)	0.76, 0.39

Computer programs: *CrysAlis PRO* (Agilent, 2013 ▸), *SHELXS97*, *SHELXL97* and *XP* in *SHELXTL* (Sheldrick, 2008 ▸).

## supplementary crystallographic information

### Crystal data

**Table:**

C_12_H_10_BrN_5_O_3_S_2_·C_3_H_7_NO	*Z* = 2
*M**_r_* = 489.38	*F*(000) = 496
Triclinic, *P*1	*D*_x_ = 1.656 Mg m^−^^3^
Hall symbol: -P 1	Mo *K*α radiation, λ = 0.71073 Å
*a* = 9.1107 (4) Å	Cell parameters from 16424 reflections
*b* = 9.9911 (4) Å	θ = 2.6–30.4°
*c* = 11.6498 (6) Å	µ = 2.34 mm^−^^1^
α = 96.482 (4)°	*T* = 100 K
β = 107.802 (4)°	Block, colourless
γ = 99.322 (4)°	0.45 × 0.40 × 0.40 mm
*V* = 981.33 (8) Å^3^	

### Data collection

**Table:**

Oxford Diffraction Xcalibur, Eos diffractometer	5844 independent reflections
Radiation source: Enhance (Mo) X-ray Source	5306 reflections with *I* > 2σ(*I*)
Graphite monochromator	*R*_int_ = 0.031
Detector resolution: 16.1419 pixels mm^-1^	θ_max_ = 30.9°, θ_min_ = 2.4°
ω–scan	*h* = −12→13
Absorption correction: multi-scan (*CrysAlis PRO*; Agilent, 2013)	*k* = −14→14
*T*_min_ = 0.824, *T*_max_ = 1.000	*l* = −16→16
52130 measured reflections	

### Refinement

**Table:**

Refinement on *F*^2^	Primary atom site location: structure-invariant direct methods
Least-squares matrix: full	Secondary atom site location: difference Fourier map
*R*[*F*^2^ > 2σ(*F*^2^)] = 0.027	Hydrogen site location: inferred from neighbouring sites
*wR*(*F*^2^) = 0.061	H atoms treated by a mixture of independent and constrained refinement
*S* = 1.06	*w* = 1/[σ^2^(*F*_o_^2^) + (0.0248*P*)^2^ + 0.6489*P*] where *P* = (*F*_o_^2^ + 2*F*_c_^2^)/3
5844 reflections	(Δ/σ)_max_ = 0.001
268 parameters	Δρ_max_ = 0.76 e Å^−^^3^
0 restraints	Δρ_min_ = −0.39 e Å^−^^3^

### Special details

**Table:**

Geometry. All e.s.d.'s (except the e.s.d. in the dihedral angle between two l.s. planes) are estimated using the full covariance matrix. The cell e.s.d.'s are taken into account individually in the estimation of e.s.d.'s in distances, angles and torsion angles; correlations between e.s.d.'s in cell parameters are only used when they are defined by crystal symmetry. An approximate (isotropic) treatment of cell e.s.d.'s is used for estimating e.s.d.'s involving l.s. planes.Non-bonded contact:3.4582 (0.0010) Br1 - O1_$6Operator for generating equivalent atoms: $6 - *x* + 2, -*y* + 2, -*z* + 1============================================================================Least-squares planes (*x*,*y*,*z* in crystal coordinates) and deviations from them (* indicates atom used to define plane)8.0547 (0.0765) *x* - 4.9253 (0.0915) *y* + 0.8579 (0.2093) *z* = 4.7396 (0.0731)* 0.0000 (0.0001) C2 * 0.0000 (0.0001) H02 * 0.0000 (0.0000) H03 0.0352 (0.0108) N4Rms deviation of fitted atoms = 0.00007.4457 (0.0028) *x* - 5.9260 (0.0044) *y* + 1.6748 (0.0062) *z* = 4.4689 (0.0045)Angle to previous plane (with approximate e.s.d.) = 7.74 (1.41)* -0.0578 (0.0009) N1 * 0.0522 (0.0009) C2 * 0.0025 (0.0009) N3 * -0.0485 (0.0010) C4 * 0.0394 (0.0010) C5 * 0.0122 (0.0009) C6 0.1986 (0.0021) N4 - 0.2565 (0.0018) S1 - 0.3373 (0.0028) C7 0.1276 (0.0023) C8 0.2186 (0.0028) N5 0.0409 (0.0018) O1 - 0.0581 (0.0020) N2Rms deviation of fitted atoms = 0.04116.0229 (0.0041) *x* - 2.3534 (0.0057) *y* + 5.9679 (0.0059) *z* = 4.9873 (0.0043)Angle to previous plane (with approximate e.s.d.) = 31.72 (0.06)* -0.0028 (0.0010) C11 * 0.0004 (0.0010) C12 * 0.0025 (0.0010) C13 * -0.0031 (0.0010) C14 * 0.0007 (0.0010) C15 * 0.0023 (0.0010) C16 - 0.0142 (0.0020) Br1 0.0055 (0.0019) S2Rms deviation of fitted atoms = 0.0022
Refinement. Refinement of *F*^2^ against ALL reflections. The weighted *R*-factor *wR* and goodness of fit *S* are based on *F*^2^, conventional *R*-factors *R* are based on *F*, with *F* set to zero for negative *F*^2^. The threshold expression of *F*^2^ > σ(*F*^2^) is used only for calculating *R*-factors(gt) *etc*. and is not relevant to the choice of reflections for refinement. *R*-factors based on *F*^2^ are statistically about twice as large as those based on *F*, and *R*- factors based on ALL data will be even larger.

### Fractional atomic coordinates and isotropic or equivalent isotropic displacement parameters (Å^2^)

**Table:**

	*x*	*y*	*z*	*U*_iso_*/*U*_eq_	
S1	0.74640 (5)	0.49517 (4)	0.94893 (3)	0.02271 (8)	
S2	0.64492 (4)	0.41433 (3)	0.34915 (3)	0.01389 (7)	
Br1	0.903097 (18)	1.043895 (15)	0.333551 (15)	0.02167 (5)	
N1	0.78069 (13)	0.40042 (11)	0.57992 (10)	0.0118 (2)	
C2	0.70629 (16)	0.30239 (14)	0.62952 (12)	0.0132 (2)	
N2	0.77711 (15)	0.36204 (12)	0.45978 (10)	0.0130 (2)	
H01	0.863 (2)	0.3749 (19)	0.4545 (17)	0.020 (5)*	
N3	0.69682 (14)	0.33056 (12)	0.74159 (11)	0.0151 (2)	
C4	0.77293 (17)	0.45373 (14)	0.80858 (12)	0.0147 (3)	
C5	0.87008 (16)	0.55054 (14)	0.77173 (12)	0.0142 (2)	
C6	0.87526 (15)	0.52734 (13)	0.65034 (12)	0.0122 (2)	
C7	0.6131 (2)	0.34218 (18)	0.95215 (15)	0.0254 (3)	
H7A	0.5211	0.3215	0.8774	0.038*	
H7B	0.5787	0.3573	1.0236	0.038*	
H7C	0.6669	0.2646	0.9572	0.038*	
C8	0.96232 (18)	0.67418 (15)	0.85179 (13)	0.0186 (3)	
N4	0.64153 (15)	0.17814 (13)	0.56513 (12)	0.0169 (2)	
H02	0.597 (2)	0.119 (2)	0.5990 (18)	0.024 (5)*	
H03	0.633 (2)	0.159 (2)	0.4900 (19)	0.025 (5)*	
N5	1.03702 (19)	0.77174 (15)	0.91924 (14)	0.0298 (3)	
O1	0.95091 (12)	0.60446 (10)	0.60406 (9)	0.01553 (19)	
O2	0.50567 (12)	0.40373 (11)	0.38292 (10)	0.0196 (2)	
O3	0.64611 (14)	0.33408 (11)	0.23994 (9)	0.0215 (2)	
C11	0.71531 (16)	0.58768 (14)	0.34507 (12)	0.0140 (2)	
C12	0.68353 (17)	0.69127 (15)	0.41854 (13)	0.0162 (3)	
H12	0.6237	0.6690	0.4703	0.019*	
C13	0.74062 (17)	0.82758 (15)	0.41502 (13)	0.0175 (3)	
H13	0.7206	0.8999	0.4646	0.021*	
C14	0.82751 (17)	0.85737 (15)	0.33814 (13)	0.0167 (3)	
C15	0.86007 (17)	0.75425 (15)	0.26524 (13)	0.0179 (3)	
H15	0.9202	0.7767	0.2137	0.021*	
C16	0.80332 (17)	0.61764 (15)	0.26891 (13)	0.0162 (3)	
H16	0.8244	0.5454	0.2199	0.019*	
N6	0.33893 (15)	0.04126 (13)	0.14664 (11)	0.0182 (2)	
O4	0.50814 (13)	0.02165 (11)	0.32929 (10)	0.0198 (2)	
C17	0.48080 (18)	0.04576 (14)	0.22375 (13)	0.0174 (3)	
H17	0.5681	0.0692	0.1963	0.021*	
C18	0.2020 (2)	0.0087 (2)	0.18574 (17)	0.0322 (4)	
H18A	0.1941	−0.0837	0.2071	0.048*	
H18B	0.1066	0.0117	0.1191	0.048*	
H18C	0.2132	0.0762	0.2574	0.048*	
C19	0.3164 (2)	0.08261 (18)	0.02765 (15)	0.0274 (3)	
H19A	0.4184	0.1036	0.0146	0.041*	
H19B	0.2700	0.1646	0.0248	0.041*	
H19C	0.2456	0.0075	−0.0366	0.041*	

### Atomic displacement parameters (Å^2^)

**Table:**

	*U*^11^	*U*^22^	*U*^33^	*U*^12^	*U*^13^	*U*^23^
S1	0.0325 (2)	0.02259 (18)	0.01318 (16)	0.00060 (15)	0.01114 (15)	0.00105 (13)
S2	0.01596 (16)	0.01541 (15)	0.01050 (14)	0.00078 (12)	0.00523 (12)	0.00418 (11)
Br1	0.02029 (8)	0.01729 (7)	0.02730 (9)	0.00028 (5)	0.00798 (6)	0.00829 (6)
N1	0.0146 (5)	0.0130 (5)	0.0090 (5)	0.0013 (4)	0.0062 (4)	0.0024 (4)
C2	0.0140 (6)	0.0143 (6)	0.0120 (6)	0.0018 (5)	0.0050 (5)	0.0048 (5)
N2	0.0151 (6)	0.0159 (5)	0.0107 (5)	0.0033 (4)	0.0077 (4)	0.0034 (4)
N3	0.0178 (6)	0.0167 (5)	0.0110 (5)	0.0015 (4)	0.0058 (4)	0.0033 (4)
C4	0.0160 (6)	0.0179 (6)	0.0106 (6)	0.0041 (5)	0.0046 (5)	0.0037 (5)
C5	0.0155 (6)	0.0141 (6)	0.0124 (6)	0.0023 (5)	0.0040 (5)	0.0018 (5)
C6	0.0105 (6)	0.0124 (6)	0.0141 (6)	0.0032 (5)	0.0038 (5)	0.0034 (5)
C7	0.0280 (8)	0.0315 (8)	0.0170 (7)	−0.0022 (6)	0.0117 (6)	0.0051 (6)
C8	0.0223 (7)	0.0173 (6)	0.0167 (7)	0.0042 (5)	0.0067 (6)	0.0044 (5)
N4	0.0226 (6)	0.0150 (5)	0.0125 (6)	−0.0014 (5)	0.0075 (5)	0.0026 (4)
N5	0.0357 (8)	0.0210 (7)	0.0259 (7)	0.0004 (6)	0.0043 (6)	0.0005 (5)
O1	0.0151 (5)	0.0149 (4)	0.0189 (5)	0.0013 (4)	0.0091 (4)	0.0051 (4)
O2	0.0147 (5)	0.0246 (5)	0.0205 (5)	0.0017 (4)	0.0069 (4)	0.0091 (4)
O3	0.0318 (6)	0.0194 (5)	0.0113 (5)	−0.0003 (4)	0.0077 (4)	0.0014 (4)
C11	0.0146 (6)	0.0153 (6)	0.0132 (6)	0.0028 (5)	0.0047 (5)	0.0062 (5)
C12	0.0161 (6)	0.0194 (6)	0.0163 (6)	0.0053 (5)	0.0076 (5)	0.0075 (5)
C13	0.0175 (7)	0.0187 (7)	0.0176 (7)	0.0058 (5)	0.0061 (5)	0.0045 (5)
C14	0.0140 (6)	0.0166 (6)	0.0182 (7)	0.0012 (5)	0.0032 (5)	0.0071 (5)
C15	0.0171 (7)	0.0212 (7)	0.0179 (7)	0.0027 (5)	0.0083 (5)	0.0084 (5)
C16	0.0173 (7)	0.0199 (7)	0.0129 (6)	0.0040 (5)	0.0063 (5)	0.0051 (5)
N6	0.0199 (6)	0.0184 (6)	0.0158 (6)	0.0022 (5)	0.0054 (5)	0.0049 (5)
O4	0.0222 (5)	0.0175 (5)	0.0161 (5)	−0.0018 (4)	0.0036 (4)	0.0044 (4)
C17	0.0203 (7)	0.0134 (6)	0.0174 (7)	−0.0009 (5)	0.0071 (5)	0.0016 (5)
C18	0.0204 (8)	0.0526 (11)	0.0288 (9)	0.0110 (8)	0.0106 (7)	0.0167 (8)
C19	0.0315 (9)	0.0315 (8)	0.0177 (7)	0.0026 (7)	0.0059 (6)	0.0101 (6)

### Geometric parameters (Å, º)

**Table:**

S1—C4	1.7392 (14)	C15—C16	1.389 (2)
S1—C7	1.8036 (16)	N6—C17	1.320 (2)
S2—O3	1.4292 (11)	N6—C19	1.4551 (19)
S2—O2	1.4305 (11)	N6—C18	1.455 (2)
S2—N2	1.6707 (13)	O4—C17	1.2373 (18)
S2—C11	1.7580 (14)	N2—H01	0.80 (2)
Br1—C14	1.8920 (14)	C7—H7A	0.9800
N1—C2	1.3801 (16)	C7—H7B	0.9800
N1—N2	1.3979 (15)	C7—H7C	0.9800
N1—C6	1.4150 (17)	N4—H02	0.86 (2)
C2—N4	1.3167 (18)	N4—H03	0.85 (2)
C2—N3	1.3361 (17)	C12—H12	0.9500
N3—C4	1.3342 (18)	C13—H13	0.9500
C4—C5	1.3971 (19)	C15—H15	0.9500
C5—C6	1.4235 (18)	C16—H16	0.9500
C5—C8	1.4235 (19)	C17—H17	0.9500
C6—O1	1.2281 (16)	C18—H18A	0.9800
C8—N5	1.147 (2)	C18—H18B	0.9800
C11—C12	1.392 (2)	C18—H18C	0.9800
C11—C16	1.3918 (19)	C19—H19A	0.9800
C12—C13	1.387 (2)	C19—H19B	0.9800
C13—C14	1.391 (2)	C19—H19C	0.9800
C14—C15	1.388 (2)		
			
C4—S1—C7	101.66 (7)	C17—N6—C18	119.55 (13)
O3—S2—O2	121.50 (7)	C19—N6—C18	118.46 (13)
O3—S2—N2	103.18 (6)	O4—C17—N6	124.64 (14)
O2—S2—N2	106.00 (6)	N1—N2—H01	111.8 (14)
O3—S2—C11	107.29 (6)	S2—N2—H01	113.5 (14)
O2—S2—C11	109.08 (7)	S1—C7—H7A	109.5
N2—S2—C11	109.23 (6)	S1—C7—H7B	109.5
C2—N1—N2	116.88 (11)	H7A—C7—H7B	109.5
C2—N1—C6	122.54 (11)	S1—C7—H7C	109.5
N2—N1—C6	120.02 (11)	H7A—C7—H7C	109.5
N4—C2—N3	118.74 (12)	H7B—C7—H7C	109.5
N4—C2—N1	119.66 (12)	C2—N4—H02	117.5 (13)
N3—C2—N1	121.60 (12)	C2—N4—H03	121.9 (14)
N1—N2—S2	117.24 (9)	H02—N4—H03	120.2 (19)
C4—N3—C2	117.70 (12)	C13—C12—H12	120.5
N3—C4—C5	123.85 (13)	C11—C12—H12	120.5
N3—C4—S1	117.37 (10)	C12—C13—H13	120.3
C5—C4—S1	118.77 (11)	C14—C13—H13	120.3
C4—C5—C6	119.61 (12)	C14—C15—H15	120.6
C4—C5—C8	121.71 (13)	C16—C15—H15	120.6
C6—C5—C8	118.67 (12)	C15—C16—H16	120.3
O1—C6—N1	119.46 (12)	C11—C16—H16	120.3
O1—C6—C5	126.95 (13)	O4—C17—H17	117.7
N1—C6—C5	113.59 (11)	N6—C17—H17	117.7
N5—C8—C5	177.93 (16)	N6—C18—H18A	109.5
C12—C11—C16	121.64 (13)	N6—C18—H18B	109.5
C12—C11—S2	119.54 (10)	H18A—C18—H18B	109.5
C16—C11—S2	118.82 (11)	N6—C18—H18C	109.5
C13—C12—C11	118.95 (13)	H18A—C18—H18C	109.5
C12—C13—C14	119.35 (13)	H18B—C18—H18C	109.5
C15—C14—C13	121.82 (13)	N6—C19—H19A	109.5
C15—C14—Br1	119.39 (11)	N6—C19—H19B	109.5
C13—C14—Br1	118.79 (11)	H19A—C19—H19B	109.5
C14—C15—C16	118.90 (13)	N6—C19—H19C	109.5
C15—C16—C11	119.35 (13)	H19A—C19—H19C	109.5
C17—N6—C19	121.65 (13)	H19B—C19—H19C	109.5
			
N2—N1—C2—N4	2.44 (19)	C4—C5—C6—O1	177.72 (13)
C6—N1—C2—N4	−168.95 (13)	C8—C5—C6—O1	−1.0 (2)
N2—N1—C2—N3	−176.97 (12)	C4—C5—C6—N1	−2.16 (18)
C6—N1—C2—N3	11.6 (2)	C8—C5—C6—N1	179.16 (12)
C2—N1—N2—S2	100.31 (12)	O3—S2—C11—C12	160.71 (11)
C6—N1—N2—S2	−88.07 (13)	O2—S2—C11—C12	27.34 (13)
O3—S2—N2—N1	−167.13 (9)	N2—S2—C11—C12	−88.10 (12)
O2—S2—N2—N1	−38.42 (11)	O3—S2—C11—C16	−19.99 (13)
C11—S2—N2—N1	78.98 (11)	O2—S2—C11—C16	−153.36 (11)
N4—C2—N3—C4	175.25 (13)	N2—S2—C11—C16	91.20 (12)
N1—C2—N3—C4	−5.3 (2)	C16—C11—C12—C13	0.3 (2)
C2—N3—C4—C5	−4.6 (2)	S2—C11—C12—C13	179.59 (11)
C2—N3—C4—S1	174.66 (10)	C11—C12—C13—C14	0.2 (2)
C7—S1—C4—N3	−0.12 (13)	C12—C13—C14—C15	−0.6 (2)
C7—S1—C4—C5	179.14 (12)	C12—C13—C14—Br1	179.51 (11)
N3—C4—C5—C6	8.4 (2)	C13—C14—C15—C16	0.4 (2)
S1—C4—C5—C6	−170.82 (10)	Br1—C14—C15—C16	−179.69 (11)
N3—C4—C5—C8	−172.98 (14)	C14—C15—C16—C11	0.1 (2)
S1—C4—C5—C8	7.81 (19)	C12—C11—C16—C15	−0.5 (2)
C2—N1—C6—O1	172.81 (12)	S2—C11—C16—C15	−179.77 (11)
N2—N1—C6—O1	1.68 (19)	C19—N6—C17—O4	174.49 (15)
C2—N1—C6—C5	−7.29 (18)	C18—N6—C17—O4	1.3 (2)
N2—N1—C6—C5	−178.42 (11)		

### Hydrogen-bond geometry (Å, º)

**Table:**

*D*—H···*A*	*D*—H	H···*A*	*D*···*A*	*D*—H···*A*
N2—H01···O1^i^	0.80 (2)	2.00 (2)	2.7784 (15)	165.5 (19)
N4—H02···O4^ii^	0.86 (2)	1.98 (2)	2.8382 (16)	177.9 (19)
N4—H03···O4	0.85 (2)	2.07 (2)	2.8005 (16)	143.1 (18)
C12—H12···O2^iii^	0.95	2.46	3.4096 (18)	173
C7—H7*B*···O3^iv^	0.98	2.45	3.2848 (19)	143
C17—H17···Br1^v^	0.95	3.05	3.6686 (15)	124

Symmetry codes: (i) −*x*+2, −*y*+1, −*z*+1; (ii) −*x*+1, −*y*, −*z*+1; (iii) −*x*+1, −*y*+1, −*z*+1; (iv) *x*, *y*, *z*+1; (v) *x*, *y*−1, *z*.
